# Balancing surface adsorption and diffusion of lithium-polysulfides on nonconductive oxides for lithium–sulfur battery design

**DOI:** 10.1038/ncomms11203

**Published:** 2016-04-05

**Authors:** Xinyong Tao, Jianguo Wang, Chong Liu, Haotian Wang, Hongbin Yao, Guangyuan Zheng, Zhi Wei Seh, Qiuxia Cai, Weiyang Li, Guangmin Zhou, Chenxi Zu, Yi Cui

**Affiliations:** 1 College of Materials Science and Engineering, Zhejiang University of Technology, Hangzhou 310014, China; 2 Department of Materials Science and Engineering, Stanford University, Stanford, California 94305, USA; 3 College of Chemical Engineering, Zhejiang University of Technology, Hangzhou 310014, China; 4 Stanford Institute for Materials and Energy Science, SLAC National Accelerator Laboratory, Menlo Park, California 94025, USA

## Abstract

Lithium–sulfur batteries have attracted attention due to their six-fold specific energy compared with conventional lithium-ion batteries. Dissolution of lithium polysulfides, volume expansion of sulfur and uncontrollable deposition of lithium sulfide are three of the main challenges for this technology. State-of-the-art sulfur cathodes based on metal-oxide nanostructures can suppress the shuttle-effect and enable controlled lithium sulfide deposition. However, a clear mechanistic understanding and corresponding selection criteria for the oxides are still lacking. Herein, various nonconductive metal-oxide nanoparticle-decorated carbon flakes are synthesized via a facile biotemplating method. The cathodes based on magnesium oxide, cerium oxide and lanthanum oxide show enhanced cycling performance. Adsorption experiments and theoretical calculations reveal that polysulfide capture by the oxides is via monolayered chemisorption. Moreover, we show that better surface diffusion leads to higher deposition efficiency of sulfide species on electrodes. Hence, oxide selection is proposed to balance optimization between sulfide-adsorption and diffusion on the oxides.

## Abstract (web summary)

Metal oxides can suppress detrimental polysulfide shuttling in lithium-sulfur batteries, however selection criteria for oxide materials are still lacking. Here, the authors investigate polysulfide adsorption and diffusion on metal oxides and propose selection criteria based on balancing these two effects.

## Graphical abstract

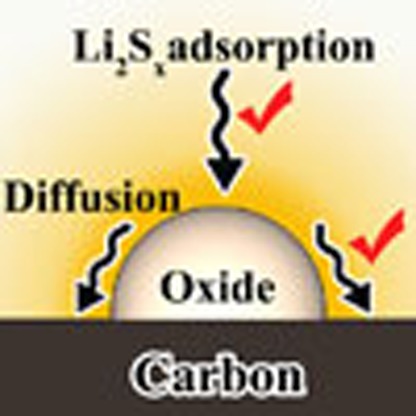

Rechargeable lithium–sulfur (Li–S) batteries have recently become one of the more exciting energy storage systems due to the low-cost and high-specific energy of sulfur cathodes^1^,^2^,^3^,^4^,^5^,^6^,^7^,^8^,^9^,^10^,^11^,^12^,^13^,^14^,^15^,^16^,^17^,^18^,^19^,^20^,^21^,^22^,^23^,^24^,^25^,^26^,^27^. Although there have been significant developments for designing state-of-the-art Li–S batteries in the past two decades, the practical application is still hindered by many material challenges, including dissolution of intermediate lithium polysulfides (Li_2_S_*x*_, *x*>3) in the electrolyte^28^, large volumetric expansion (80%) of sulfur upon lithiation^6^, and poor electronic/ionic conductivity of sulfur and lithium sulfide (Li_2_S) (ref. 6). To date, tremendous efforts have been made to solve the above problems by constructing advanced composite cathode materials. One effective strategy is the encapsulation of sulfur to prevent the leakage of active materials and suppress the shuttle effect of high-order Li_2_S_*x*_ (refs 3, 6). Oxides^6^, carbon^3^,^29^, polymers^30^ and metals^31^ are proved to be good matrix materials for the encapsulation of sulfur^19^. The second approach is the controllable deposition of the discharge product Li_2_S, which is an ionic and electronic insulator^4^. The detaching and irreversible phase transformation of Li_2_S is considered as the main reason for capacity fading^4^,^32^. The third strategy is using Li_2_S as a starting cathode material, which undergoes volumetric contraction instead of the expansion in the case of sulfur^20^. In addition, Li_2_S-based cathodes can be paired with lithium metal-free anodes such as graphite, silicon and alloys^33^, thus suppressing the dendrite growth and the corresponding safety concerns of lithium-metal anodes.

All the previous research reveals the importance of understanding the sulfide species interaction with the matrix materials. Our earlier work pointed out that the usual carbon substrates interact with Li_2_S_*x*_ weakly but the polar group enabled strong interaction with Li_2_S_*x*_, which can facilitate the Li_2_S_*x*_ trapping and promote the attachment of solid Li_2_S_2_ and Li_2_S and improve the cycling stability of Li–S batteries^34^. Many similar examples followed-up using polymers^35^,^36^, oxides^4^,^19^, sulfides^20^, functionalized graphene^18^,^26^,^27^,^37^,^38^, metal organic framework^39^ and nitrogen doped carbon^36^,^40^, which all have polar surfaces to adsorb Li_2_S_*x*_ species. Results from this study seem to suggest that the stronger the binding, the better the Li–S batteries. In addition, it was recognized that using conducting materials such as indium tin oxide^4^ and Ti_4_O_7_ (ref. 19) is preferable due to the electron transfer needed to induce electrochemical reaction. Our recent work showed that indium tin oxide decorated carbon nanofibres can enhance the redox kinetics of Li_2_S_*x*_, realize the controllable deposition of Li_2_S and improve the electrochemical performance of Li–S batteries^4^.

Besides the conductive matrix material, there are indeed abundant insulating materials to trap Li_2_S_*x*_. But there is a fundamental problem here: insulated materials cannot transport electrons. Trapping Li_2_S_*x*_ on insulating materials would cause them to be accumulated in electronically inactive areas and reduce the capacity retention. However, some studies have shown improvements of the electrochemical properties after the decoration of the electrode with poor conductive oxides such as MnO_2_ (ref. 16), Mg_0.6_Ni_0.4_O (ref. 12), Al_2_O_3_ (ref. 41) and La_2_O_3_ (ref. 42).

The above background research has motivated us to hypothesize that surface diffusion of Li_2_S_*x*_ species on solid substrates can play an important role in Li–S battery electrochemical performance. This is particularly important for insulating solid materials with strong adsorption of Li_2_S_*x*_. The competition between the adsorption and diffusion of the Li_2_S_*x*_ adsorbates on solid substrates can be very important, yet has been overlooked for Li–S batteries.

Usually, the nonconductive metal oxides work together with the carbon matrix to improve the conductivity of sulfur cathodes. For the modified carbon matrix with some nonconductive metal oxides nanostructures (Fig. 1), there is no direct electron transfer between these oxides and Li_2_S_*x*_ species. Because of the poor conductivity of the metal oxide, the absorbed Li_2_S_*x*_ should be transferred from the surface of the oxide to the conductive carbon substrate to undergo the electrochemical reaction. Therefore the competitive surface diffusion and adsorption of sulfur species must play key roles in the Li–S batteries. If the metal oxide has weak Li_2_S_*x*_ capture capability (Fig. 1a), a large amount of Li_2_S_*x*_ can diffuse away from the carbon matrix, resulting in serious shuttle effect and uncontrollable deposition of Li_2_S. When the diffusion of sulfur species from the surface of oxide to carbon is difficult (Fig. 1c), the electrochemical reaction of Li_2_S_*x*_ and the corresponding growth of Li_2_S on the oxide/carbon is impeded. Therefore, the balance optimization between Li_2_S_*x*_ adsorption and diffusion on the metal oxides surface is necessary (Fig. 1b).

In this work, various nonconductive metal oxide (MgO, Al_2_O_3_, CeO_2_, La_2_O_3_ and CaO) nanoparticles-decorated carbon flakes are synthesized via a facile and generic biotemplating method using Kapok trees fibres as both the template and the carbon source. The sulfur cathodes based on MgO, CeO_2_ and La_2_O_3_ show higher capacity and better cycling stability. Adsorption test, microstructure analysis and electrochemical performance evaluation combined with density functional theory (DFT) calculations reveal that better surface diffusion leads to higher deposition efficiency of sulfide species. A comprehensive-oxide-selection criteria referring to the strong binding, high surface area and good surface diffusion properties is proposed.

## Results

### Sulfide capture by metal oxides

**Figure 2:**
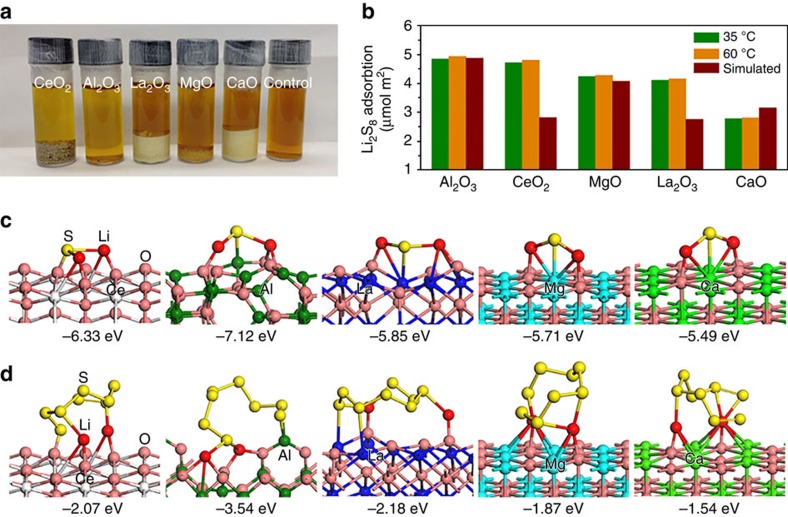
Adsorption test and relative models of sulfide species on the surface of metal oxides. (**a**) Digital images of the Li_2_S_8_ trapping by the metal oxide nanoparticles in DOL/DME (1:1, v-v) solution. (**b**) Experimental and simulated adsorption amount of Li_2_S_8_ on different metal oxides. The simulated adsorption was based on the monolayer adsorption model. (**c**) Optimized geometries of the most stable Li_2_S on CeO_2_(111), Al_2_O_3_(110), La_2_O_3_(001), MgO(100) and CaO(100) surfaces. (**d**) Optimized geometries of most stable Li_2_S_8_ on the metal oxide surface.

To reveal the role of metal oxides in Li–S batteries, five kinds of pure metal oxide-nanoparticles were prepared by a generic Pechini sol–gel method^13^. 1,3-dioxolane (DOL) and dimethoxyethane (DME) are commonly used solvent in the Li–S battery electrolyte^4^. Therefore, 0.005 M Li_2_S_8_ in DOL/DME (1:1, v-v) was prepared for the adsorption test of sulfides. Figure 2a shows the camera image of the adsorption test using different mass of oxide samples with the same total surface area. The colour of the solution containing Al_2_O_3_ and CeO_2_ is lighter than the others, indicating better adsorption of these two metal oxide nanoparticles. Inductively coupled plasma-optical emission spectroscopy (ICP-OES) results reveal that Al_2_O_3_ and CaO show the biggest and the smallest absorption capability, respectively (Fig. 2b). In addition, it was found that the adsorption capacity increases slightly with the rise of temperature (Fig. 2b and Supplementary Table 1), which is one essential characteristic of chemisorption. The measured Li_2_S_8_ adsorption quantity of metal oxides is in the range of 2.78–4.94 μmol m^−2^, close to the simulated monolayer adsorption capacity ranging from 2.76 to 4.88 μmol m^−2^ (Fig. 2b). Therefore, the Li_2_S_8_ capture involves monolayer adsorption, which is another well-known characteristic of chemisorption.

In order to better understand the absorption mechanism, DFT calculation was performed to reveal the corresponding adsorption energy and sites (Fig. 2c,d and Supplementary Figs 2–6). Considering both low-order and high-order, Li_2_S_*x*_ are important discharge products of Li–S batteries, we choose both Li_2_S and Li_2_S_8_ as the prototype for modelling. Figure 2c shows the optimized geometries of the most stable Li_2_S on CeO_2_(111), Al_2_O_3_(110), La_2_O_3_(001), MgO(100) and CaO(100) surfaces. On the entire surface, the most favourable binding site of Li_2_S is two Li atoms bonding with the oxygen atom of metal oxide (Fig. 2c). On Al_2_O_3_(110), the Li of Li_2_S is the bridge site of two oxygen atoms, on other four metal oxides, the Li is on the atop site of oxygen. On MgO(100) CaO(100) and La_2_O_3_(001) surfaces, the sulfur of Li_2_S is away from the metal oxides surface. Sulfur is bonding with oxygen on CeO_2_(111), in which the sulfur–oxygen distance is 1.70 Å while with Al on Al_2_O_3_(110), in which the Al–S distance is 2.21 Å. The adsorption energy of Li_2_S on CeO_2_(111), Al_2_O_3_(110), La_2_O_3_(001), MgO(100) and CaO(100) surfaces is −6.33, −7.12, −0.5.85, −5.71 and −5.49 eV, respectively. The optimized stable configurations of Li_2_S_8_ on five different surfaces are also shown in Fig. 2d and Supplementary Figs 2–6. Although the Li of Li_2_S_8_ has similar bonding sites on the metal oxide surface as that of Li_2_S, the optimized most stable configuration of Li_2_S_8_ on each metal oxide is quite different. A lot of initial geometries of Li_2_S_8_ on each metal oxide have been considered in our calculations. It is found that after optimization, the structures have big change due to the interaction between Li_2_S_8_ and metal oxides. For both Li_2_S and Li_2_S_8_ on metal oxides, the bonding between Li and oxygen plays a major role. In addition, only two or three sulfur atoms of Li_2_S_8_ are bonding with the oxide surface. The adsorption energies of Li_2_S_8_ have similar trend on these metal surfaces with Li_2_S, while are much weaker than Li_2_S. The calculated adsorption energies of both Li_2_S and Li_2_S_8_ are in agreement with the experimental adsorption test results of Li_2_S_8_ on the oxide nanoparticles (Fig. 2b and Supplementary Table 1).

### Biotemplated fabrication of oxides/carbon nanostructures

**Figure 3:**
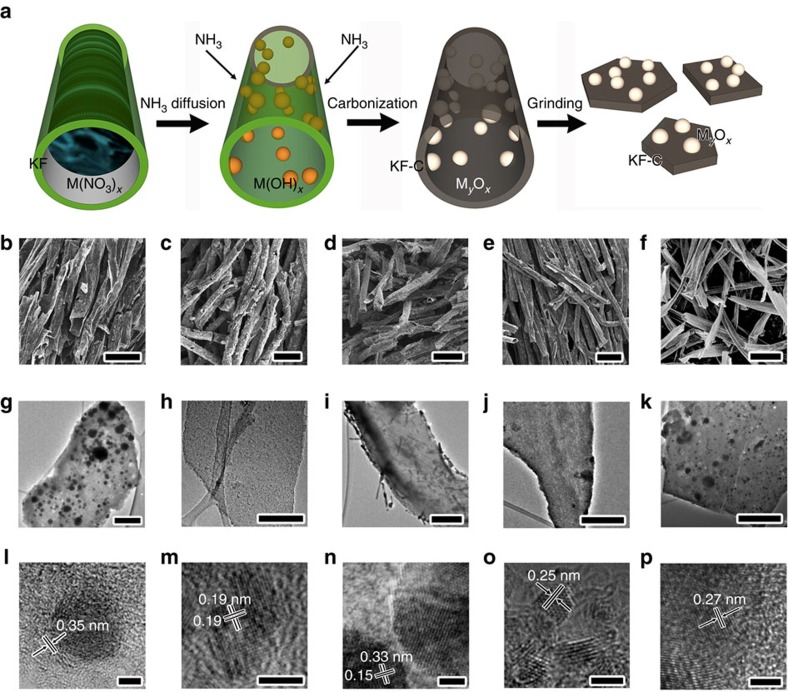
Fabrication and microstructures of oxides/carbon nanostructures. (**a**) Schematic illustration of synthesis of oxides/carbon using the kapok tree fibres (KF) as both template and carbon sources. (**b**–**f**) SEM images of Al_2_O_3_/C, CeO_2_/C, La_2_O_3_/C, MgO/C and CaO/C composites, respectively (scale bar=50 μm). (**g**–**k**) The corresponding TEM images of Al_2_O_3_/C, CeO_2_/C, La_2_O_3_/C, MgO/C and CaO/C, respectively (scale bar=300 nm). (**l**–**p**) The corresponding HRTEM images of Al_2_O_3_/C, CeO_2_/C, La_2_O_3_/C, MgO/C and CaO/C, respectively (scale bar=3 nm).

Although most of these oxides have remarkable adsorption behaviour for sulfide species, they are poor electronic conductors. Conductivity is one of the most important factors affecting the performance of Li–S batteries. Therefore, we fabricated metal-oxides (MgO, Al_2_O_3_, CeO_2_, La_2_O_3_ and CaO) nanoparticles anchored on porous carbon nanoflakes to form an electronic conductive oxide/carbon nanocomposite. To fabricate the nanocomposite, Kapok fibres (KFs)^43^ were used as both the template and the carbon source (Fig. 3a). KFs are low-cost and high-yield agriculture products derived from the fruits of Kapok tree, which is chemically composed of 64% cellulose, 13% lignin and 23% pentosan^43^. In addition, the KFs have unique hollow lumens with a thin wall thickness ≤1 μm, which enables good sorption capacity through capillary force. Therefore, metal nitrate solution can be easily absorbed into the KFs (Fig. 3a). When the NH_3_ gas diffuses into the KFs through the porous cell wall and the open end, metal hydroxides nanoparticles will be formed on the surface of the cell wall due to the confinement effect of KF template. After drying at 90 °C for 12 h, carbonization at 850 °C for 1.5 h and the following facile grinding, carbon nanoflakes decorated with metal oxide nanoparticles can be obtained (Fig. 3a). Figure 3b–f shows the scanning electron microscopy (SEM) images of Al_2_O_3_/C, CeO_2_/C, La_2_O_3_/C, MgO/C and CaO/C nanocomposites, respectively. The composite remains the macromorphology of the original Kapok tree fibres, which have a unique hollow structure with a large lumen and a thin fibre wall. After simple grinding, the delicate carbon microtubes are converted to carbon nanoflakes (Fig. 3g–k and Supplementary Figs 7–9).

Moreover, transmission electron microscopy (TEM) images show that abundant oxide nanostructures are located on the carbon matrix (Fig. 3g–k). Figure 3l is the representative high resolution TEM (HRTEM) image of the Al_2_O_3_/C sample, showing the amorphous structure of Al_2_O_3_ and partially graphitized structure of C. The interlayer spacing is about 0.35 nm, corresponding to (002) planes of graphite. Figure 3m shows the [001] zone axis HRTEM image of a typical CeO_2_ nanoparticle in CeO_2_/C composite. The lattice spacing of 0.19 nm in Fig. 3m can be attributed to {220} crystal planes of the face centred cubic (fcc) phase CeO_2_. The TEM image of La_2_O_3_/C is showed in Fig. 3i, suggesting that abundant rod-shaped nanoparticles are distributed in the carbon matrix. The corresponding HRTEM image in Fig. 3n shows that the La_2_O_3_ particle is single crystalline. The lattice spacings (0.15 and 0.33 nm) and the interplanar angle match the finger print of (004) and (100) planes of hexagonal phase La_2_O_3_. HRTEM image of MgO/C sample in Fig. 3o indicates lattice fringes with regular spacing of 0.25 nm, which can be indexed to (111) planes of fcc MgO. Figure 3p shows the HRTEM image of CaO/C sample. The fringes with spacing of 0.27 nm is corresponding to (111) planes of fcc CaO. These TEM results indicate that the Kapok tree fibre can act as ideal and general template for the synthesis of oxide nanostructures due to the confinement effect of fibre substrate.

### Electrochemical performance of composite cathodes

**Figure 4:**
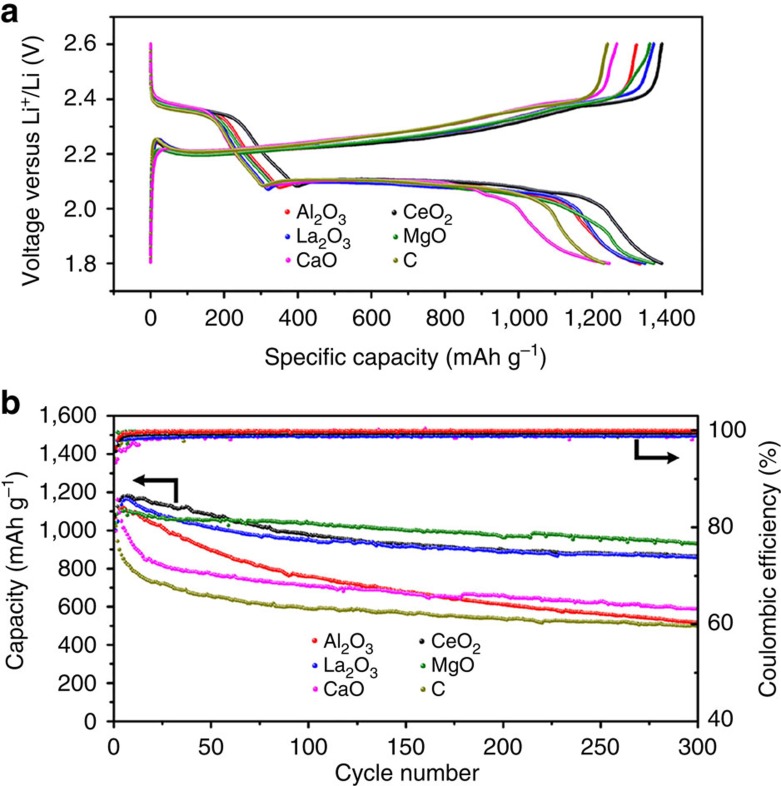
Charge–discharge curves and cycling performance of the sulfur composite electrodes. (**a**) Representative charge–discharge profiles of the composite electrodes based on different oxide/carbon nanostructures at 0.1 C. (**b**) Specific capacity and the corresponding Coulombic efficiency of the composite electrodes upon prolonged 300 charge–discharge cycles at 0.5 C.

Thermal diffusion method was used to fabricate the sulfur/M_*x*_O_*y*_/C composite^19^. The mass loading of the electrode ranges from 0.7 to 1.2 mg cm^−2^. The electrolyte was 1 M Lithium bis(trifluoromethanesulphonyl)imide in DOL and DME, with LiNO_3_ as additive to passivate the lithium anode. Figure 4a shows the representative charge–discharge curves of the composite electrode based on different oxide/carbon nanostructures at a current rate of 0.1 C (1 C=1,672 mA g^−1^). All the discharge curves show two typical discharge plateaus at 2.35 and 2.10 V, which can be assigned to the formation of high-order and low-order Li_2_S_*x*_, respectively^9^. No obvious difference can be found for the potential of the discharge plateaus. However, the CaO/C and C composite cathodes show higher charge over-potentials than those of CeO_2_/C, La_2_O_3_/C, Al_2_O_3_/C and MgO/C composite electrodes (Fig. 4a). The specific discharge capacities of Al_2_O_3_/C, CeO_2_/C, La_2_O_3_/C, MgO/C, CaO/C and C at 0.1 C rate are measured to be 1,330, 1,388, 1,345, 1,368, 1,246 and 1,230 mAh g^−1^, respectively (Fig. 4a). It can be found that the CaO/C and C composite cathodes show relatively lower discharge capacity. The high over-potential and low discharge capacity may result from the serious dissolution of Li_2_S_*x*_ in electrolyte (shuttle effect), which causes the active material loss and the increase of electrolyte viscosity. ICP-OES test (Supplementary Table 2) based on the same mass of Al_2_O_3_/C, CeO_2_/C, La_2_O_3_/C, MgO/C, CaO/C and C samples reveal that both CaO/C and C have poorer Li_2_S_*x*_ capture capability compared with other samples.

Besides the specific capacity, cycling performance is one of the most important characteristics for Li–S batteries. Therefore, all the cathodes were subject to prolonged cycling. Figure 4b shows the discharge capacity and the corresponding Coulombic efficiency of the cathodes upon prolonged 300 cycles at 0.5 C. The representative charge/discharge curves (Fig. 4a) and the cycling performance (Fig. 4b) indicate that Al_2_O_3_/C, CeO_2_/C, La_2_O_3_/C and MgO/C composite electrode show high specific capacity in the first several cycles. However, the composite electrodes show distinct capacity retention capability. The Al_2_O_3_/C, CaO/C and C cathodes exhibit obvious capacity fading especially in the first 100 cycles. Compared with Al_2_O_3_/C, CaO/C and C cathodes, the electrode based on CeO_2_/C, La_2_O_3_/C and MgO/C show better cycling performance and the MgO/C cathode is the best among all the samples. The capacity decay per cycle is 0.171, 0.066, 0.047, 0.034, 0.136 and 0.170% for Al_2_O_3_/C, CeO_2_/C, La_2_O_3_/C, MgO/C, CaO/C and C, respectively. Considering the serious capacity, decay mainly happens in the first 50 cycles, the average Columbic efficiency in the first 100 cycles was calculated (Fig. 4b). Al_2_O_3_/C, CeO_2_/C, La_2_O_3_/C, MgO/C, CaO/C and C cathodes show 99.6, 99.1, 98.7, 99.4, 98.3 and 98.8% columbic efficiency. Lower Columbic efficiency of Li–S batteries resulted from the significant Li_2_S_*x*_dissolution, which cause the loss of S material and shuttle effect^19^. This can be also supported by the ICP-OES results, revealing that Al_2_O_3_/C has the best capture capability and CaO/C and C have poorer capture capability for Li_2_S_8_(Supplementary Table 2). Although Al_2_O_3_/C cathodes possess high initial discharge capacity and good Columbic efficiency, the rate of capacity decay is higher than those of CeO_2_/C, La_2_O_3_/C and MgO/C cathodes. The distinct Columbic efficiency for Al_2_O_3_ (99.6%) cathode from both CaO/C (98.3%) and C (98.8%) cathodes may imply different capacity decay mechanism.

### Analysis of capacity failure mechanism of composite cathodes

**Figure 5:**
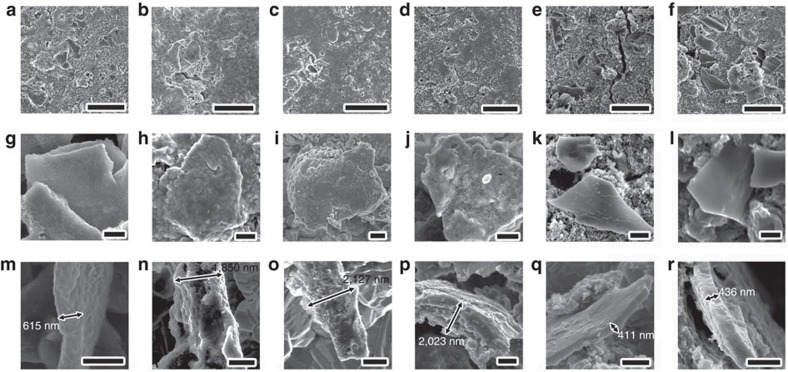
Morphology of the discharged composite electrodes after cycling. (**a**–**f**) SEM images of the cycled composite electrodes based on Al_2_O_3_/C, CeO_2_/C, La_2_O_3_/C, MgO/C, CaO/C and carbon nanostructures, respectively. (**g**–**l**) Top view and (**m**–**r**) cross-sectional SEM images show typical Al_2_O_3_/C, CeO_2_/C, La_2_O_3_/C, MgO/C, CaO/C and C nanostructures after cycling, respectively. Scale bars=10μm (**a**–**f**) and 1 μm (**g**–**r**).

In order to further reveal the detailed capacity decay mechanism, some batteries were disassembled after 100 cycles at 0.5 C to observe the morphology evolution of the cathode materials by SEM. Figure 5a–f shows low magnification SEM images of the cycled electrodes based on Al_2_O_3_/C, CeO_2_/C, La_2_O_3_/C, MgO/C, CaO/C and C nanostructures, respectively. In contrast to CeO_2_/C (Fig. 5b), La_2_O_3_/C (Fig. 5c) and MgO/C (Fig. 5d) electrodes with uniform and flat surface, the Al_2_O_3_/C (Fig. 5a), CaO/C (Fig. 5e) and C (Fig. 5f) cathodes show high surface roughness. Some carbon nanoflakes with well-defined profile can be observed on the surface of the Al_2_O_3_/C (Fig. 5a), CaO/C (Fig. 5e) and C (Fig. 5f) cathodes after 100 cycles. In addition, some cracks and pinholes can be found in the CaO/C and carbon cathodes, which may result from the significant dissolution and loss of sulfur. Figure 5g–l and m–r shows the top-view and cross-sectional SEM images of cycled Al_2_O_3_/C, CeO_2_/C, La_2_O_3_/C, MgO/C, CaO/C and C cathodes, respectively. Only a thin, uniform and dense Li_2_S film can be found on the surface of Al_2_O_3_/C (Fig. 5g,m). Abundant Li_2_S particles with irregular shape were formed between the Al_2_O_3_/C nanoflakes (Supplementary Fig. 1). Similar phenomenon can also be found in the CaO/C (Fig. 5k,q) and C (Fig. 5l,r) cathodes. Some characteristic stripes (Fig. 5k,l) and distinct fracture surface (Fig. 5q,r) indicate that the Li_2_S particles are detached from the oxides/C matrix and may become electrochemically inactive. This is due to the uncontrollable precipitation of Li_2_S on the non-polar or weakly polar surface^6^, which leads the further decay of capacity. In contrast, it is not easy to identify the oxide/carbon nanoflakes from the CeO_2_/C (Fig. 5h), La_2_O_3_/C (Fig. 5i) and MgO/C (Fig. 5j) cathodes. To obtain the cross-sectional morphology of the cathodes, the electrode materials were scraped from the Al foil current collector and mounted on a copper foil with rough surface for SEM observation. CeO_2_/C (Fig. 5n), La_2_O_3_/C (Fig. 5o) and MgO/C (Fig. 5p) nanoflakes were wrapped by thick Li_2_S layer. Supposing that the average thickness of carbon nanoflakers is 450 nm, the thickness of Li_2_S layer deposited on the surface of CeO_2_/C, La_2_O_3_/C and MgO/C will be 1,400, 1,677 and 1,573 nm, respectively. After mechanical scrapping and mounting processes, no detachment can be observed, indicating that there is good cohesion between the Li_2_S layer and the oxide/C nanoflakes. These results are consistent with our DFT calculation results in Fig. 2. Because the Li_2_S is a poor ionic and electronic conductor, the good combination of Li_2_S with the conductive matrix must be favourable for the reversible electrochemical reaction in the following charging process^4^. Therefore, the better cycling performance of CeO_2_/C, La_2_O_3_/C and MgO/C cathodes can be attributed to the controllable precipitation of Li_2_S on the polar surface of carbon matrix.

By now, some important questions arise: why Li_2_S shows different deposition behaviour on various oxide/C surfaces? Which kind of oxide/C surface is favourable for the controllable precipitation of Li_2_S? Because the nucleation and initial growth sites of Li_2_S are located on the surface of oxide/C, the growth behaviour of Li_2_S must be related to surface chemical properties of oxide/C matrix. First, the oxide/C surface should absorb high-order Li_2_S_*x*_ owing to its strong adsorption ability (Fig. 2d), which acts as the sulfur source for the growth of Li_2_S. The absorbed Li_2_S_*x*_ must be transferred from the oxide surface to conductive carbon surface to enable the electrochemical reactions due to the insulating properties of these metal oxides. Therefore, the distribution and structure of Li_2_S will be affected by the surface diffusion properties of Li_2_S_*x*_ species on the oxide/C substrate.

### Lithium ion diffusion properties and mechanism of the cathodes

**Figure 6:**
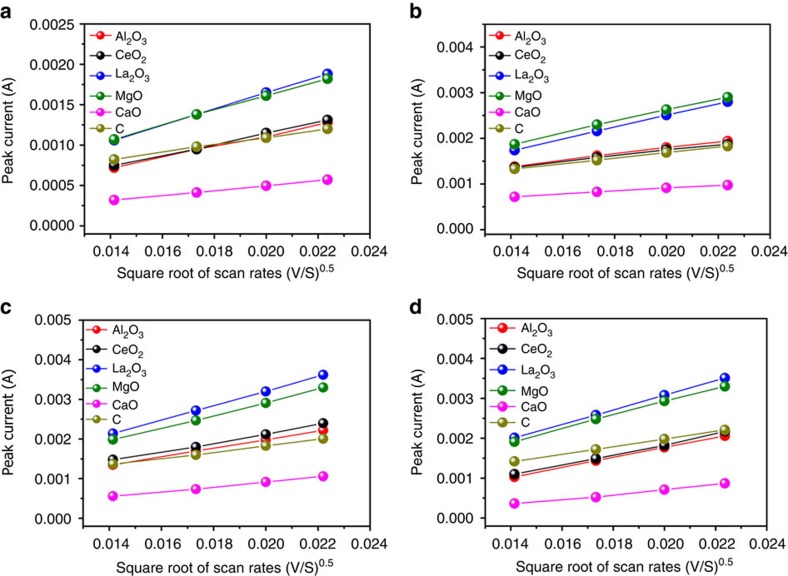
Lithium ion diffusion properties of the electrode at various voltage scan rates. Plot of CV peak current of (**a**) the cathodic reaction 1 (S_8_→Li_2_S_4_), (**b**) the cathodic reaction 2 (Li_2_S_4_→Li_2_S), (**c**) the anodic reaction 1 (Li_2_S→Li_2_S_4_) and (**d**) the anodic reaction 2 (Li_2_S_4_→S_8_) versus the square root of scan rates.

Although it is very difficult to get the diffusivity of Li_2_S_*x*_ on the oxide surface from the electrochemical measurement, the lithium diffusivity in the whole Li–S batteries can offer the important information about Li_2_S_*x*_ surface diffusion because of that the most favourable binding site of sulfides is two Li atoms bonding with metal oxide (Fig. 2c,d). In order to explore the lithium diffusion properties, we performed cyclic voltammetry (CV) measurements under different scanning rates ranging from 0.2 to 0.5 mV s^−1^. As shown in Fig. 6, all cathodic and anodic peak currents are linear with the square root of scan rates, from which the lithium diffusion performance can be estimated using the classical Randles Sevcik equation^44^:


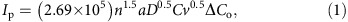


where, *I*_p_ is the peak current, *n* is the number of electrons per reaction species, *a* is the active electrode area, *D* is the lithium ion diffusion coefficient, Δ*C*_o_ is the Li concentration change corresponding to the electrochemical reaction. The *n*, *a* and Δ*C*_o_ are constant in our battery system. The slopes of curves in Fig. 6a–d are positively correlated to the corresponding lithium ion diffusion, which indicates that the sulfur composite cathode based on both CaO/C and C have lower diffusivity. Abundant high viscosity Li_2_S_*x*_ dissolved in the electrolyte and the poor Li_2_S_*x*_ capture capability of both CaO/C and C is believed to be the main reason for their low diffusivity. Compared with CaO/C, CeO_2_/C, Al_2_O_3_/C and C electrodes, La_2_O_3_/C and MgO/C samples show better diffusion properties and the measured diffusivity of Al_2_O_3_/C cathode is comparable with the CeO_2_/C.

**Figure 7:**
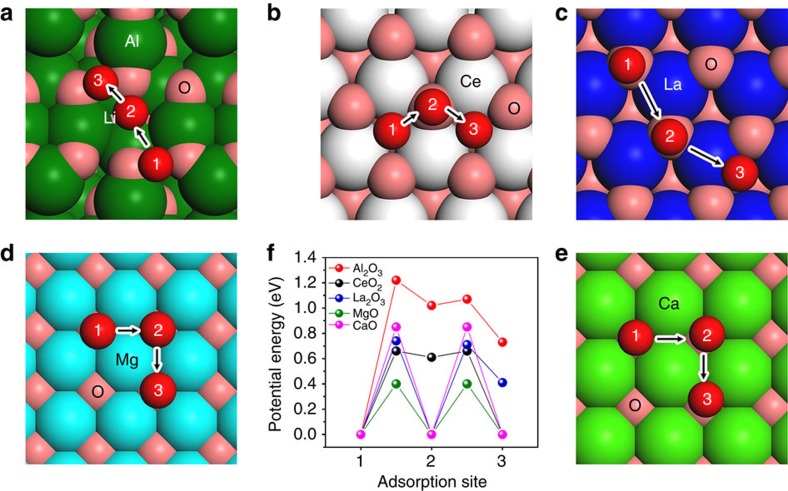
Lithium diffusion mechanism on the surface of various metal oxides. (**a**–**e**) Minimum energy path for lithium ion diffusion on Al_2_O_3_(110), CeO_2_(111), La_2_O_3_(001), MgO(100) and CaO(100) surfaces, respectively. (**f**) Potential energy profiles for Li^+^ diffusion along different adsorption sites on the oxide surface.

The diffusion of lithium on the surface of various metal oxides has been investigated by the DFT calculation (Fig. 7). Because the most favourable binding site of sulfides is two Li atoms bonding with metal oxide (Fig. 2c,d), the calculated Li ion diffusion can also indicate the diffusivity of sulfides species on the surface of the oxide. On MgO(100), CaO(100) and La_2_O_3_(001) surfaces, the diffusions of Li in different dimensions can be realized on three equivalent adsorption sites (Fig. 7). Among the three kinds of surfaces, the diffusion barrier of Li on CaO(100) is largest. The space group of MgO is same with CaO, however, the diffusion barrier of Li on MgO(100) is about 0.45 eV lower than that on CaO(100). The suitable adsorption energies of sulfide species and small diffusion barriers of Li on MgO will lead to the formation of abundant Li_2_S particles on MgO/C surfaces, which are responsible for the best cycling performance of MgO/C cathodes. On CeO_2_(111) surfaces, the large diffusion barrier is 0.66 eV, which is similar with that on La_2_O_3_(001) surfaces. This may explain why CeO_2_/C and La_2_O_3_/C cathodes show similar cycling performance. Among the five kinds of metal oxides surfaces, the largest diffusion barrier (1.22 eV) of Li is found to be on Al_2_O_3_(110), which is about three times of that on MgO(100). It is seen that sulfide species can strongly adsorb, however, difficult to diffuse on Al_2_O_3_. Although Al_2_O_3_ has the strongest Li_2_S_8_ adsorption (Fig. 2), the slow diffusion of Li_2_S_*x*_ indicated that Al_2_O_3_ may not be a good additive for sulfur cathode.

## Discussion

Based on our experimental results, we clarify three functions of these oxides. The first basic function of these metal oxides is the Li_2_S_*x*_ adsorption. Although many literatures have reported the Li_2_S_*x*_ capture capacities, the detailed absorption mechanism is still unclear. Our DFT calculation and temperature swing adsorption experiments (Fig. 2) confirm that the monolayer chemisorption is dominant during the Li_2_S_*x*_ capture. The second function of these metal oxides, especially some nonconductive oxides, is the Li_2_S_*x*_ transfer station, which transports the Li_2_S_*x*_ from the poorly conductive oxide surface to high conductive carbon matrix to ensure the full electrochemical conversion. The third function is to induce the controlled growth of Li_2_S species on the surface of the composite instead of random deposition. Many reports proved that the uncontrolled deposition will result in electrochemically inactive large agglomerations of Li_2_S. The subsequent detachment of Li_2_S from the oxide/carbon matrix is the main capacity decay mechanism, which can be supported by the SEM observation in Fig. 5. The SEM observations, diffusion test and DFT calculations revealed that the deposition of Li_2_S on the surface of oxide/carbon matrix may be influenced by the lithium ion diffusion properties on the surface of metal oxides, which has not yet been identified. Surface diffusion properties will affect the distribution and growth of Li_2_S.

Based on these functions of nonconductive metals oxides, we can propose an oxide selection criterion for the Li–S batteries. Because the first role of oxides is adsorption, the binding between the sulfides species and the matrix should be strong, which can both suppress the shuttle effect and enable the full utilization of active materials. Considering that the Li_2_S_*x*_ capture is the monolayer chemisorption and the adsorption amount will depend on the surface area of oxides, uniformly distributed oxides nanostructures with high surface area are essential. Although strong binding and high surface area are preconditions, the surface diffusion properties of oxides are also very important, which affect the distribution and structure of Li_2_S. An optimized balance between Li_2_S_*x*_ adsorption and surface diffusion is favourable for the sulfide species to deposit on the surface of oxide/carbon matrix, keep active during the cycling and ensure the final good cycling performance of batteries. In addition, some other factors such as electric conductivities, chemical stability and lithiation/delithiation of the oxides also need to be considered.

In conclusion, a series of nonconductive metal oxides (MgO, Al_2_O_3_, CeO_2_, La_2_O_3_ and CaO) nanoparticles anchored on porous carbon nanoflakes have been synthesized successfully via a facile and generic biotemplating method using Kapok trees fibres as both the template and the carbon source. The composite cathode materials based on the MgO/C, La_2_O_3_/C and CeO_2_/C nanoflakes show higher capacity and better cycling performance. Moreover, the working mechanisms of these oxides were revealed by adsorption test, microstructure analysis, electrochemical performance evaluation and DFT calculations. In addition, the comprehensive oxide selection criteria referring to the strong binding, high surface area and good surface diffusion properties were proposed for the first time. We believe that our proposed selection criteria can be generalized to other matrix materials for high performance Li–S batteries such as metal sulfides, metal nitrides, metal chlorides, and so on.

## Methods

### Preparation of metal oxide nanoparticles

A generic Pechini sol–gel method^13^ was used to synthesize the pure metal oxides. 0.01 mol metal nitrates of the desired metals were dissolved in 5 ml of deionized water under stirring. 0.015 mol citric acid was then added in to the prepared solution to chelate the metal ions and heated to 200 °C to form a dry and porous gel. After the calcination of the gel at 850 °C for 1 h, pure metal oxide (Al_2_O_3_, CeO_2_, La_2_O_3_, MgO and CaO) nanoparticles were obtained.

### Biotemplated fabrication of metal oxide/carbon nanoflakes

Owing to the poor electric conductivity of Al_2_O_3_, CeO_2_, La_2_O_3_, MgO and CaO, a series or metal oxide decorated porous carbon flakes were fabricated using the Kapok tree fibres as both the template and the carbon sources. To start with, 0.01 mol metal nitrates of the desired metals were dissolved in 40 ml of deionized water to form transparent solution. Commercial Kapok tree fibre was washed with acetone and ethanol to remove the surface wax and dipped in to the nitrate solution. After 2 h soaking, the nitrate loaded fibres were separated from the solution and introduced into ammonia atmosphere to ensure the formation of the metal hydroxides, followed by the drying at 100 °C for 10 h to remove the water. Then the dried fibres were inserted into the tube furnace and calcined at 850 °C for 1 h with 100 sccm continuous flow of argon. After cooling, the obtained carbon microtubes remaining the macromorphology of original Kapok tree fibres were easily grinded into carbon nanoflakes decorated with metal oxide nanoparticles.

### Adsorption test

To prepare Li_2_S_8_ solution, stoichiometric Li_2_S and sulfur were dissolved in 1,3-dioxolane/1,2-dimethoxyethane solution (1:1 in volume) and stirred at 80 °C for 24 h. Then the Li_2_S_8_ solution was diluted to 0.005 M for the capture test. Before the adsorption test, all the oxide and oxide/carbon samples were dried under vacuum at 80 °C for 24 h. After the immersion of the samples in 0.005 M Li_2_S_8_ at different temperatures, 20 μl of the solution was transferred for the ICP-OES test^45^.

### Characterization

The morphology and the microstructure were studied using SEM (FEI, XL30 Sirion) and TEM (FEI, Tecnai G2 F20 X-TWIN). The specific surface area was characterized from nitrogen adsorption–desorption measurement (Micromeritics, ASAP 2020). ICP-OES was conducted using a Thermo Scientific ICAP 6300 Duo View Spectrometer.

### Electrochemical measurements

The sulfur cathode materials were prepared via a facile thermal diffusion method^19^. First, sulfur and grinded oxide/carbon nanoflakes with a weight ratio of 1:3 were mixed with appropriate amount of CS_2_ solution. After the evaporation of CS_2_, the mixture were pressed and heated at 155 °C for 12 h under vacuum to obtain the composite cathode materials. To fabricate the 2,023 type coin cells for the electrochemical measurements, the synthesized composites were grinded again and mixed with conductive carbon black and polyvinylidene fluoride binder in N-methyl-2-pyrrolidinone (70:20:10 by weight) to form a slurry. After a 12 h magnetic stirring, the slurry was coated onto the aluminium foil and dried overnight at 60 °C under vacuum. The sulfur content was in the range of 63–70 wt% and the mass loading of the electrodes ranges from 0.7 to 1.2 mg cm^−2^. 1 M lithium bis(trifluoromethanesulphonyl)imide dissolved in a mixture of 1,3-dioxolaneand dimethoxymethane (1:1 by volume) with 0.1 M LiNO_3_ additive was used as the electrolyte. Galvanostatic cycling was performed on Arbin or MTI testers with the potential range of 1.8–2.6 V versus Li/Li^+^ at ambient temperature.

### DFT calculations

All calculations were performed using the Vienna *ab initio* Simulation Package code^46^ based on density functional theory. The projector augmented wave potentials were used to describe the interaction between ions and electrons^47^,^48^. Nonlocal exchange correlation energy was evaluated using the Perdew-Burke-Ernzehof functional. The electron wave function was expanded using plane waves with an energy cutoff of 400 eV. All structures were optimized with force convergence criterion of 10 meV Å^-1^. All oxide surfaces were created based on the corresponding optimized bulk unit cell, which were in good agreement with the experimental values. A 4 × 4 × 1 Monkhorst–Pack k-point mesh and a vacuum slab of about 15 Å was inserted between the surface slabs for all the metal oxide models. The cell parameter is 8.40 × 8.07 × 25.00 Å for Al_2_O_3_(110), 11.67 × 11.67 × 22.94 Å for CeO_2_(111), 11.82 × 11.82 × 25.00 Å for La_2_O_3_(001), 8.99 × 8.99 × 20.00 Å for MgO(100) and 10.25 × 10.25 × 20.00 Å for CaO(100). DFT+*U* approach, where *U* is an empirical parameter for on site electronic correlations, was used in the calculation of CeO_2_(111) with a *U* value of 5.0 eV (ref. 49). The adsorption energies (*E*_a_) for S and Li_2_S on the metal oxide surfaces are defined as *E*_a_=*E*_total_–*E*_ads_–*E*_suf_, where *E*_total_ is the total energy of the adsorbed system, *E*_ads_ is the energy of the adsorbate in vacuum and *E*_suf_ is the energy of the optimized clean surface slab.

## Additional information

**How to cite this article:** Tao, X. Y. *et al.* Balancing surface adsorption and diffusion of lithium-polysulfides on nonconductive oxides for lithium–sulfur battery design. *Nat. Commun.* 7:11203 doi: 10.1038/ncomms11203 (2016).

## Supplementary Material

Supplementary Information Supplementary Figures 1-9 and Supplementary Tables 1 & 2.

## Figures and Tables

**Figure 1:**
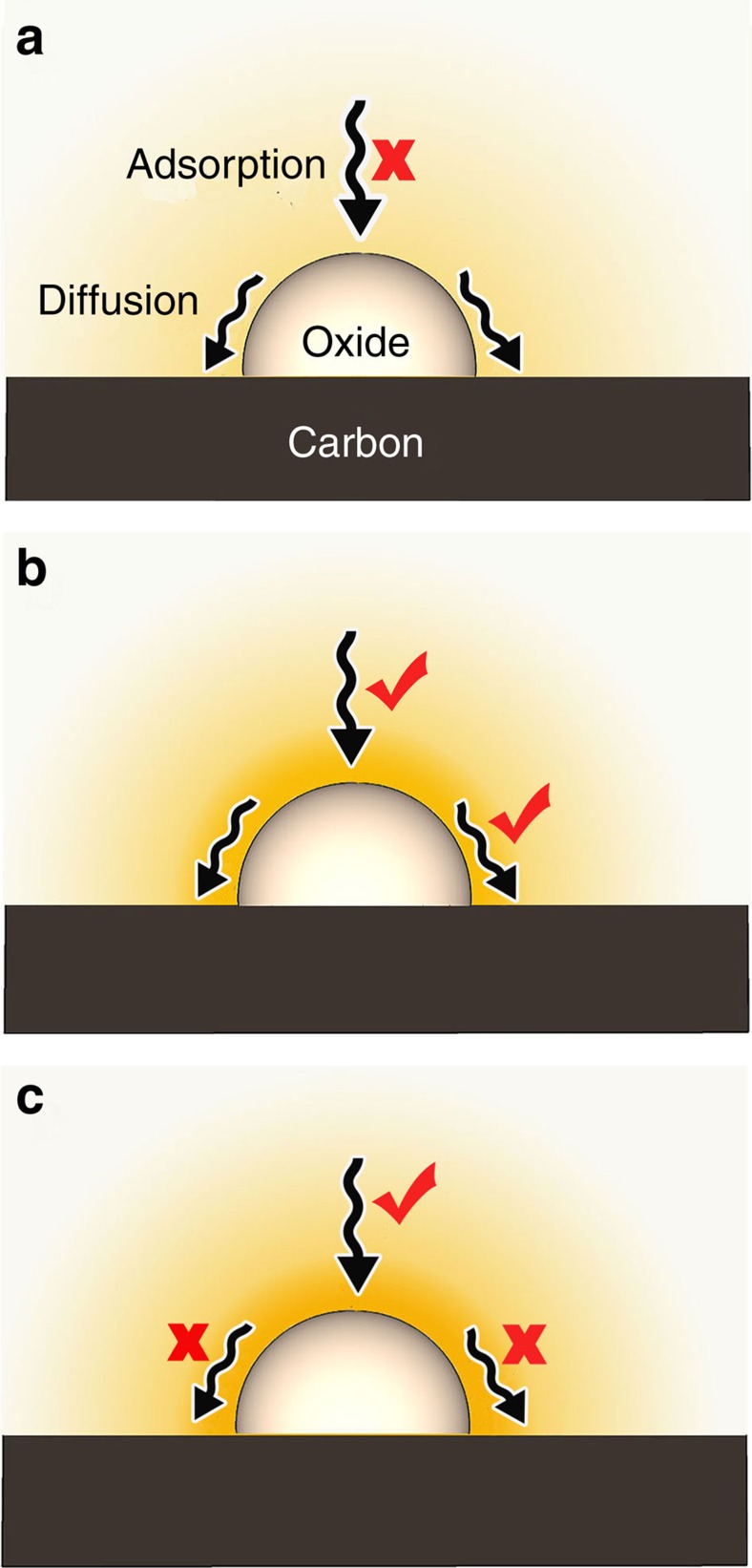
Schematic of the Li_2_S_*x*_ adsorption and diffusion on the surface of various nonconductive metal oxides. (**a**) The metal oxide with weak Li_2_S_*x*_ adsorption capability; only few Li_2_S_*x*_ can be captured by the oxide; (**b**) the metal oxide with both strong adsorption and good diffusion, which is favourable for the electrochemical reaction and the controllable deposition of sulfur species; (**c**) the metal oxide with strong bonding but without good diffusion; the growth of Li_2_S and the electrochemical reaction on the oxide/C surface is impeded.
