# Supplementary material for: Balancing surface adsorption and diffusion of lithium-polysulfides on nonconductive oxides for lithium–sulfur battery design
Source: Nat Commun. 2016 Apr 5;7:11203. doi: 10.1038/ncomms11203 (PMC4822044; doi:10.1038/ncomms11203)
Supplement: Supplementary Information — Supplementary Figures 1-9 and Supplementary Tables 1 & 2. [file ncomms11203-s1.pdf]

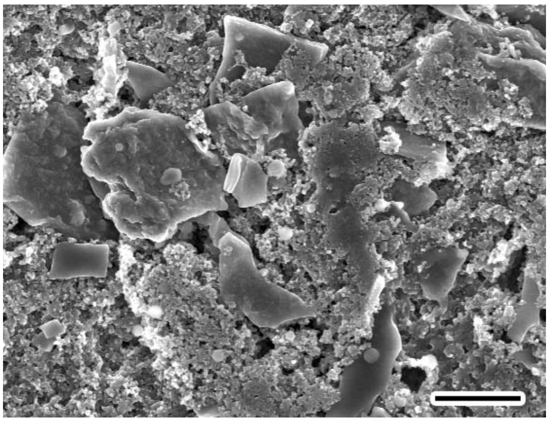

Supplementary Figure 1 | SEM image of the cycled composite electrodes based on Al_2O_3/C. Abundant Li_2S particles with irregular shape were formed between the Al_2O_3/C nanoflakes after 100 cycles. Only few Li_2S film can be deposited on the surface of Al_2O_3/C, indicating the uncontrolled deposition of Li_2S.

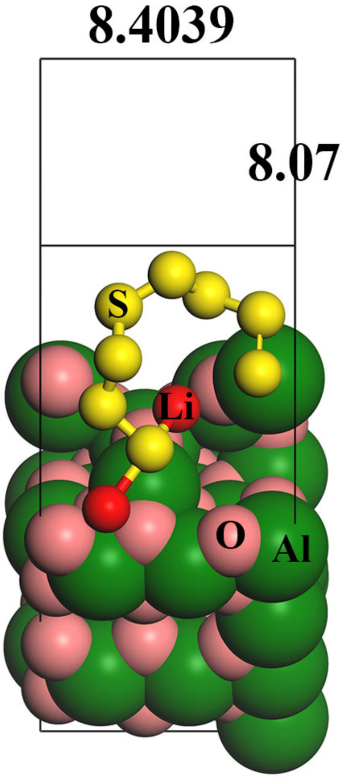

Supplementary Figure 2 | Optimized geometries of most stable Li_2S_8 on Al_2O_3(110).

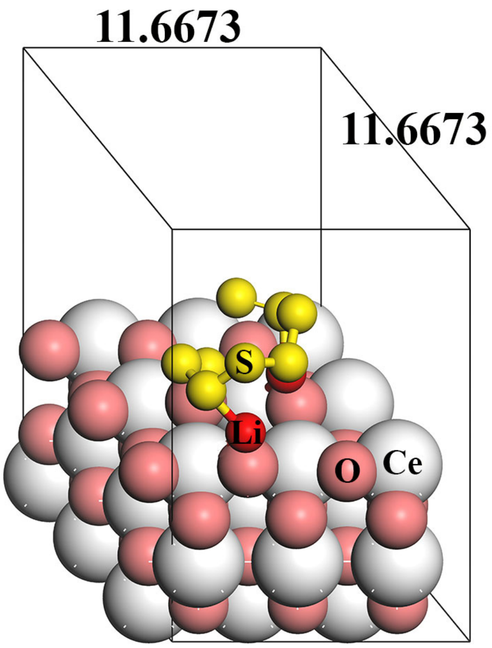

Supplementary Figure 3 | Optimized geometries of most stable Li_2S_8 on $CeO_2(111)$.

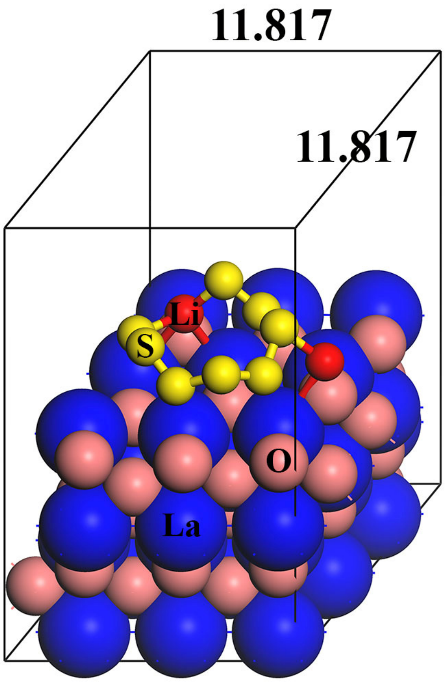

Supplementary Figure 4 | Optimized geometries of most stable Li_2S_8 on La_2O_3(001).

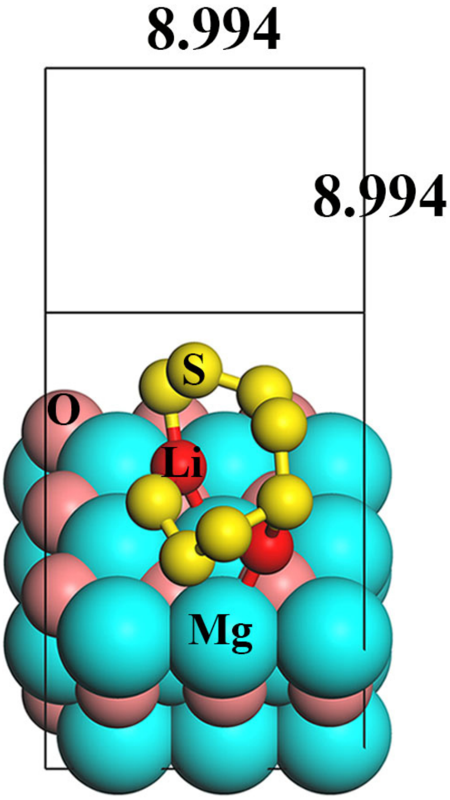

Supplementary Figure 5 | Optimized geometries of most stable Li_2S_8 on MgO(100).

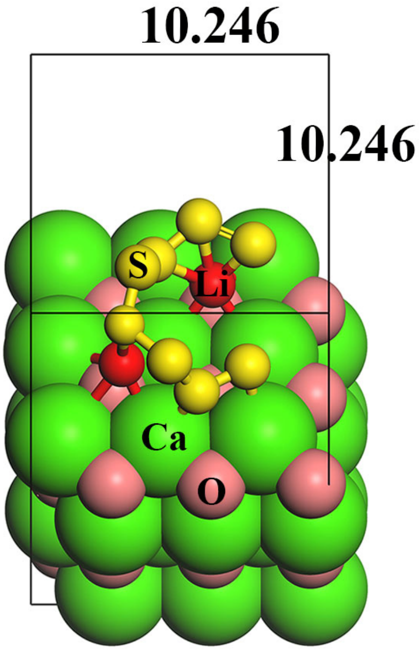

Supplementary Figure 6 | Optimized geometries of most stable Li_2S_8 on CaO(100).

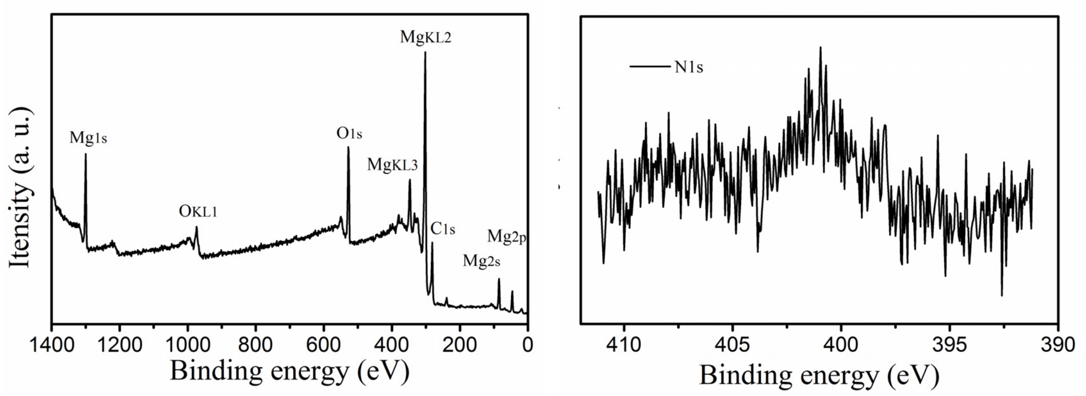

Supplementary Figure 7 | XPS characterization of MgO/C sample. Representative survey scan XPS spectrum of the MgO/C (left), indicating that the sample contains mainly Mg, O and C. No obvious N signal can be detected in the survey spectrum. The high resolution XPS spectrum for N1s of the MgO/C sample (right) reveals that trace oxygen (<0.7 wt%) can be detected. Similar phenomena were found for the other oxide/carbon samples.

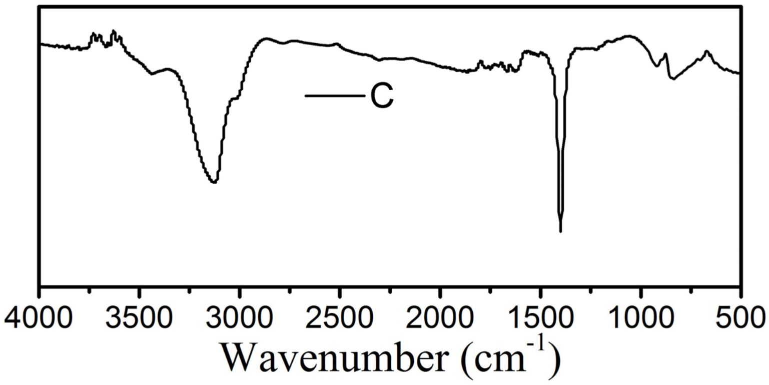

Supplementary Figure 8 | Representative FTIR spectra of pure C sample derived from Kapok fibers. The peaks at 3123 and 1380 cm^{-1} can be ascribed to the existence of –OH. Similar peaks can be observed in all oxide/carbon composite samples. Because Kapok fibers are chemically composed of 64% cellulose (($C_6H_{10}O_5$)n), 13% lignin, and 23% pentosan. Residual –OH will be remained during the pyrolysis of fibers. This kind of surface group will be beneficial to the strong bonding between the metal oxide nanoparticles and carbon matrix.

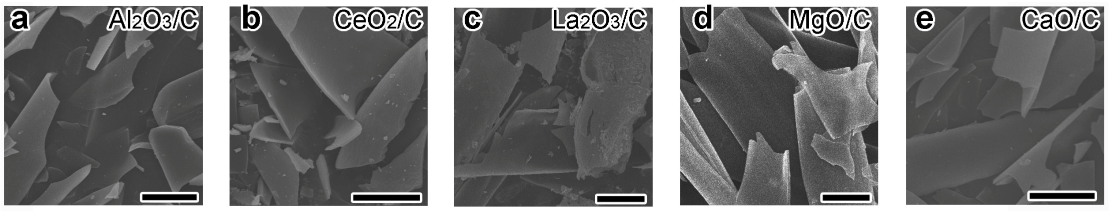

Supplementary Figure 9 | Representative SEM images of the composites after grinding. Abundant carbon flakes can be obtained after the grinding of the composite fibers (scale bar, 10 μm). No obvious difference can be found for these oxide/carbon composites. Compared with the sulfur/oxide/carbon composite (Fig. 5), the flakes in supplementary Fig. 9 have bigger size. This is because that the sulfur loaded oxide/carbon composite has been grinded again to prepare the slurry for the cathode.

Supplementary Table 1 | Li_2S_8 capture results of metal oxide nanoparticles. Adsorption percentage of Li_2S_8 (0.005M, 2 ml) by different metal oxide nanoparticles with the same total surface area (1.6 m^2) at different temperatures based on the ICP-OES test.

	Al_2O_3	CeO_2	La_2O_3	MgO	CaO
Adsorption percentage at 35 °C	77.6 %	75.5 %	65.8 %	67.9 %	44.5 %
Adsorption percentage at 60 °C	79.1 %	76.9 %	66.5 %	68.5 %	45.0 %

Supplementary Table 2 | Li_2S_8 capture results of carbon nanocomposites. Adsorption percentage of Li_2S_8 by different metal oxide/carbon nanocomposites with the same mass (0.015g) based on the ICP-OES test.

	Al_2O_3/**C**	CeO_2/**C**	La_2O_3/**C**	MgO/**C**	CaO/**C**	**C**
Adsorption percentage	83.5 %	80.1 %	64.7 %	81.2 %	55.3 %	11.1%
